# Epigenetics and early domestication: differences in hypothalamic DNA methylation between red junglefowl divergently selected for high or low fear of humans

**DOI:** 10.1186/s12711-018-0384-z

**Published:** 2018-04-02

**Authors:** Johan Bélteky, Beatrix Agnvall, Lejla Bektic, Andrey Höglund, Per Jensen, Carlos Guerrero-Bosagna

**Affiliations:** AVIAN Behavioural Physiology and Genomics Group, IFM Biology, Linköping University, 581 83 Linköping, Sweden

## Abstract

**Background:**

Domestication of animals leads to large phenotypic alterations within a short evolutionary time-period. Such alterations are caused by genomic variations, yet the prevalence of modified traits is higher than expected if they were caused only by classical genetics and mutations. Epigenetic mechanisms may also be important in driving domesticated phenotypes such as behavior traits. Gene expression can be modulated epigenetically by mechanisms such as DNA methylation, resulting in modifications that are not only variable and susceptible to environmental stimuli, but also sometimes transgenerationally stable. To study such mechanisms in early domestication, we used as model two selected lines of red junglefowl (ancestors of modern chickens) that were bred for either high or low fear of humans over five generations, and investigated differences in hypothalamic DNA methylation between the two populations.

**Results:**

Twenty-two 1-kb windows were differentially methylated between the two selected lines at *p* < 0.05 after false discovery rate correction. The annotated functions of the genes within these windows indicated epigenetic regulation of metabolic and signaling pathways, which agrees with the changes in gene expression that were previously reported for the same tissue and animals.

**Conclusions:**

Our results show that selection for an important domestication-related behavioral trait such as tameness can cause divergent epigenetic patterns within only five generations, and that these changes could have an important role in chicken domestication.

**Electronic supplementary material:**

The online version of this article (10.1186/s12711-018-0384-z) contains supplementary material, which is available to authorized users.

## Background

Animal domestication is a process that occurred in an evolutionary short timespan—less than 10,000 years [1]—and substantially altered several traits such as size, behavior, coat color, and physiological and morphological features in all domesticated species. Similar phenotypic variations tend to reoccur even in widely unrelated species, which are referred to as the ‘domestication phenotype’ [1–3]. This domestication process has altered the genetic structure that underlies these traits and has created distinct patterns that differentiate domestic animals from their wild counterparts [4–7]. Similar phenotypic changes are also seen in recent experimental domestication processes such as in the silver fox [8]. Selection for tameness, or decreased fear of humans, was undoubtedly strong during the early period of domestication and has been proposed to be the major factor that drove the domesticated phenotype, which may, accordingly, have developed partly as correlated selection responses [8–11].

Accumulating evidence shows that phenotypic variation in behavior and other traits within a species or breed can be influenced by epigenetic factors [12], in addition to genetic factors. For example, variation in personality of the great tit is statistically associated with DNA methylation levels at a dopamine receptor gene [13]. Similarly, in rat pups, the DNA methylation status of the hippocampal glucocorticoid receptor gene is affected by maternal behavior [14]. In domestic chickens, differences in DNA methylation are related to susceptibility to disease [15], immune responses [16], growth, and metabolism [17]. Moreover, epigenetic differences (e.g. in red blood cells) can emerge simply after individuals are subjected to different rearing conditions [18].

DNA methylation, i.e. the addition of a methyl group to cytosine in CpG dinucleotides, has the potential to affect gene expression. Although DNA methylation patterns are generally maintained after cell division, they can sometimes be modified by the action of external stimuli [14, 19]. Environmentally-altered DNA methylation patterns can be transmitted through the germ line [20] and be stable in somatic tissues over generations [21–25]. Somatic epigenetic differences, whether shaped by the environment or intentionally or unintentionally selected, can in turn affect phenotypic traits. Thus, epigenetic mechanisms could be an important factor in the rapid phenotypic changes that occur during domestication. This is supported, for example, by evidence of significant hypermethylation in purebred dogs compared to wolves [26], in domestic compared to wild worms [27], and in domesticated White Leghorn chickens compared to the ancestral red junglefowl (RJF) [28]. Interestingly, in the example of Darwin’s finches, DNA methylation in blood was shown to be a better indicator of evolutionary phenotypic diversification than overall genetic changes (copy number variations) [29].

The chicken, the world’s most produced species for food, was domesticated from the RJF (*Gallus gallus*) approximately 8000 years ago and is today one of the most phenotypically diverse species of vertebrates [30–32]. With both wild and domestic chickens being available, they have been used in many comparative studies that evaluated domestication effects [33–35]. These studies have, for example, shown that domesticated chickens have variable coloration patterns, grow faster, reproduce more efficiently, and differ in their social behavior and general activity compared to RJF. In the current experiment, we attempted to experimentally recreate the early domestication process of chickens by selecting RJF bi-directionally for low or high levels of fear of humans [36]. Five generations of repeated selection generated significant phenotypic variation that affected size, behavior, and metabolism [11, 36, 37], as well as transcriptomic differences in both hypothalamic and frontal cortex tissues [38, 39]. Hence, domesticated phenotypes developed in a few generations (five) as side effects of continuous selection for high or low fear of humans.

In this study, DNA samples from the hypothalamus of the fifth generation of a lineage of RJF selected for high or low fear of humans were used to perform methylated DNA immunoprecipitation (MeDIP) sequencing, in order to identify differentially-methylated (DM) regions. Previous studies on the same populations found significant differences in brain gene expression as a consequence of selection [38]. Hence, our hypothesis was that epigenetic differences, measured as differential DNA methylation, would be observed between individuals selected for high or low fear of humans over five generations. We analyzed whether these differences mainly targeted genes and genomic regions that are relevant for tameness and stress.

## Methods

### Animals and sampling

Two unrelated populations of RJF were used to generate a parental population (P0), from which two selection lines were bred for high or low fear of humans for five generations (S5). For a detailed description of the breeding scheme, selection, and housing conditions of animals, see [36, 37]. Briefly, after an outbreeding scheme that lasted for two generations, the P0 generation consisted of approximately 70 birds. The P0 generation was divided into two groups composed of animals with high or low levels of fearfulness towards humans based on scores recorded from a standardized test of fear of humans, described in detail in [36]. Individuals were then bi-directionally selected based on scores of fear of humans, resulting in two lines, i.e. one with a high fear of humans (HFH) and one with a low fear of humans (LFH). Approximately 50 animals per selection line were hatched in each generation from 5 to 10 families, and animals from the two lines were housed and reared together upon hatching in order to standardize rearing conditions. All animals received food and water ad libitum.

Animals from generation S5 were sacrificed at the age of 48 weeks by rapid decapitation. Brains were dissected from the skull and snap-frozen in liquid nitrogen within 10 min, as reported in [38]. To access the hypothalamus, which is located in the inferior region of the brain, the whole brain was dissected into smaller parts. The region between the two optic lobes was rotated upside down to reveal a white butterfly-like pattern, with the thalamus/hypothalamus centered as a small, red protruding structure. In total, 12 S5 individuals were used for this study: six HFH and six LFH birds. Each group consisted of three males and three females, which were chosen randomly from each experimental group. These same 12 individuals had previously been used in studies that examined brain transcriptomic changes [38, 39].

### DNA isolation

DNA was extracted from hypothalamus tissue using an AllPrep RNA/DNA kit (Qiagen), following the manufacturer’s instructions. In short, approximately 20 to 30 mg of tissue were homogenized with 600 µL of Buffer RTL Plus using FastPrep^®^-24 (MP Biomedicals). DNA was separated from RNA using AllPrep DNA spin columns, which were kept on ice while RNA was purified for gene expression analysis [38]. 350 µL of Buffer AW1 were added to each spin column, which was then centrifuged for 15 s at 17,000 g. The supernatant was discarded and a mixture of 20 µL of proteinase K (20 mg/mL) and 60 µL of Buffer AW1 was added to each sample. The samples were then incubated for 5 min at room temperature, cleaned with 350 µL of Buffer AW1, centrifuged for 15 s at full speed (17,000 g), and centrifuged again with 500 µL of Buffer AW2 for 2 min at full speed. Columns were dried by an additional centrifugation at 17,000 g for 1 min and then placed in 1.5 mL microcentrifuge tubes. For elution of DNA, 50 µL of Buffer EB were added to the spin column, followed by a 10-min incubation at room temperature and centrifugation for 1 min at 8000 g. DNA concentration and purity were measured using a NanoDrop^®^ ND-2000c (ThermoFisher Scientific).

### MeDIP

DNA methylation analysis was performed through enrichment of the methylated fraction of the genome by immunoprecipitation with an anti-methyl-cytosine antibody (MeDIP) [40], followed by next-generation sequencing (MeDIP-seq) [41]. MeDIP-seq is a validated method for determining DNA methylation that is widely used in epigenetic research, and shows excellent performance for genome coverage compared to equivalent methods [42]. The MeDIP procedure was carried out according to a protocol that was previously optimized in our laboratory using chicken DNA [43]. From each sample, 4 µg DNA were used for the MeDIP capture. DNA was diluted in H_2_O to a total volume of 8.0 µL, and sonicated at “high” setting for six 30-s intervals using a Bioruptor^®^ Standard sonicator (Diagenode). Samples were then run on a 2% agarose gel for confirmation of fragment lengths. Samples were cleaned from excessively long fragments using a PCR purification kit (Qiagen), following the manufacturer’s instructions, and sample concentrations were measured using a NanoDrop^®^ ND-2000c (ThermoFisher Scientific). The sonicated DNA was diluted with 450 µL of TE buffer (10 mM Tris HCl, pH 7.5; 1 mM EDTA) and heat-denatured at 95 °C for 10 min, followed by cooling on ice for 5 min. To each sample, 51 µL of 10× IP buffer (100 mM NaPhosphate, pH 7; 5 M NaCl, 250 µL Triton-X 100) were added, followed by the addition of 10 µg of antibody (monoclonal mouse anti 5-methylcytosine (2 µg/µL), 5-mC, Diagenode). Then, samples were incubated at 4 °C for 2 h on a rotating platform. Agarose beads (Protein A/G Plus-Agarose, Santa Cruz Biotechnology) were washed before use by centrifuging 50 µL of bead suspension in a 1.5 mL microcentrifuge tube for 2 min at 6000 rpm at 4 °C. The supernatant was removed and 1 mL of PBS-BSA 0.1% solution (1 mL 1× PBS + 2 µL 50 mg/ml BSA) was added. The mixture was incubated for 5 min at 4 °C on a rotating platform and then centrifuged for 2 min at 6000 rpm and 4 °C. The supernatant was subsequently removed, and the cleaning steps were repeated three more times. After cleaning, 50 µL of 1× IP buffer were added to the washed beads, and the DNA-antibody mixture was transferred to the bead mixture. The solution was incubated for 2 h at 4 °C on a rotating platform. The beads and captured DNA-antibodies were washed by centrifugation of the mixture for 2 min at 6000 rpm and 4 °C. The supernatant was removed and 1 mL of 1× IP buffer was added. The mixture was incubated for 5 min at 4 °C on a rotating platform, followed by centrifugation for 2 min at 6000 rpm and 4 °C, and removal of the supernatant. This procedure was repeated three times. To digest the beads, 210 µL of digestion buffer and 20 µL of Proteinase K (20 mg/mL) were added, followed by incubation for 2 h at 55 °C on a rotating platform. The DNA was cleaned from the bead fragments by filtering through Pierce™ Spin Cups—Paper Filter (ThermoFisher Scientific) while centrifuging at max speed for 30 s. The flow-through was collected and 3 µL of glycogen (5 mg/mL) were added. DNA was precipitated by adding 20 µL of 5 M NaCl and 750 µL ethanol, both ice cold. The solution was mixed and incubated for 30 min on ice before centrifugation at 14,000 rpm for 30 min at 4 °C. The supernatant was carefully removed and samples dried in a heating block at 50 °C for 5 min. Samples were resuspended in 30 µL of H_2_O and heated on a heating block at 50 °C for 5 min before measuring DNA concentrations on a Nanodrop. The DNA samples were then used for whole-genome amplification using a WGA2 kit (Sigma-Aldrich), following the manufacturer’s instructions. WGA samples were cleaned using a QIAquick PCR Purification Kit (Qiagen) following the manufacturer’s instructions, and used for next-generation sequencing.

### Next-generation sequencing

The 12 samples of DNA that were extracted from hypothalamus tissue from six HRH and six LFH individuals and enriched for the methylated fraction were sequenced. Sequencing was performed on IonProton chips (Ion Torrent Systems, Inc) at the National Genomics Infrastructure (NGI), using the Ion Fragment Library kit according to the manufacturer’s protocol. Data generated from the sequencing was processed using the Torrent Suite (version 5.0.2) software (ThermoFisher Scientific), which is a complete analysis solution for Ion Torrent data that includes signal processing, base calling, trimming of low-quality reads, as well as alignment to a reference genome. After quality checks, the data were aligned to the Galgal4 chicken reference genome (International Chicken Genome Consortium). Two samples of DNA enriched for the methylated fraction were loaded per Ion PI chip. Thus, we used six chips for 12 samples. The raw sequencing data were uploaded to Array Express (http://www.ebi.ac.uk/arrayexpress/) under accession number E-MTAB-6407.

### Statistical analyses

Pairwise sample comparisons of methylated regions between selection groups, sexes or the interaction between sex and selection were performed in R (version 3.3.1) using edgeR, integrated in the Bioconductor (release 3.3) package MEDIPS [44], by dividing the genome into 1-kb windows, starting at the first position on chromosome 1, and trimming away duplicated sequences. Benjamini–Hochberg correction via R’s p.adjust function was used to adjust for false discovery rate (FDR) in the multiple testing, with a cut-off of *p* < 0.1 used for significance. The cut-off was used to detect changes in a larger number of windows, allowing for a better overview of the effects of selection. In order to validate the results, random resampling was performed by randomizing samples into two random groups of six samples each, regardless of HFH or LFH selection, and running the entire MEDIPS analysis as above. The randomized groups were balanced regarding sex, to avoid sex differences in the results. Twenty replicated runs were carried out.

DM regions that covered an annotated gene region or its promoter, defined as starting 7.25 kb upstream of the transcriptional start site [28], were included in the set of regions for functional analyses. The software WebGestalt2017 (updated 1/27/2017) was used for gene ontology enrichment and KEGG pathway analysis on gene symbols obtained via Ensembl [45]. WebGestalt uses a hypergeometric distribution for significance estimation and combines it with Bonferroni–Hochberg adjustment of *p* values. All known chicken genes in the chicken genome from Ensembl were used as a base for enrichment analysis. In order to identify gene network modules that were over-represented in the DM regions, the web-based ConsensusPathDB tool was used [46] with standard settings and allowing for intermediate nodes.

## Results

### Differentially-methylated regions

A total of 430 million sequencing reads were generated, of which more than 99% were aligned to the chicken reference genome, resulting in a 9× genome coverage per sample. Each sample generated approximately 8.5 Gb of sequence data, with an average read length of 160 bp.

**Fig. 1:**
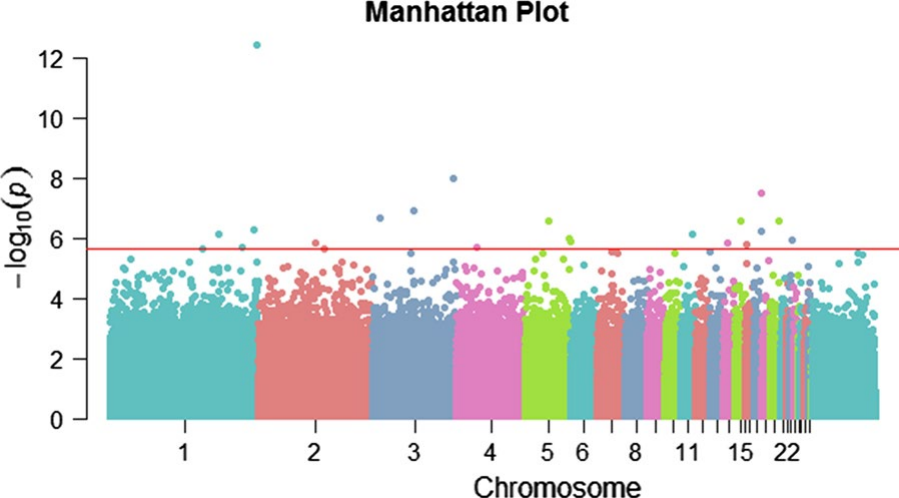
Manhattan plot of differentially-methylated (DM) windows between selection lines. All 990,000 windows were plotted with genomic location on the X-axis and negative log10 *p* values on the Y-axis. The red horizontal line indicates the threshold for significantly DM windows at *p* < 0.1 after FDR correction. Microchromosome labels were filtered out for readability

A total of 990,000 windows were analyzed by edgeR, which covered 10.6 million CpGs in the chicken autosomal genome. By sorting windows by the estimated log-transformed fold change between the HFH and LFH lines, approximately 50% of all windows were found to have a negative fold change (i.e. more methylation in the LFH than HFH samples). Moreover, the distribution of CpGs in windows with positive or negative fold changes occurred in equal proportions, which indicated that there was no bias for CpG enrichment in either of the two selection groups (Table 1). Using MEDIPS on sequence counts, 51,048 significantly differentially methylated windows were detected at *p* < 0.05, with only 22 that remained significant at *p* < 0.1 adjusted by FDR (Fig. 1 and Table 2). Nine of the 22 windows that displayed significant differential methylation were hypermethylated in the LFH group. The number of CpGs within the 1-kb windows ranged from 3 to 23, yielding a CpG density of less than 3 CpG/100 bp, which is in line with previous results obtained on somatic cells when analyzing transgenerational DM regions in response to environmental challenges in rats by MeDIP [47].

**Table 1:** Summary statistics of sequenced methylated regions in the selection lines

	Total	Positive logFC	Negative logFC
Windows	990,461	489,962	500,499
CpGs in windows	10,610,935	5,174,029	5,427,885
Significant windows	22	13	9
CpGs in significantly DM windows	187	132	55
Significant windows males	51	21	30
Significant windows females	66	30	36

Total number of windows and number of CpG dinucleotides within the windows, and differences between selection objectives for both groups and within sex. A positive log-transformed fold change (logFC) indicates windows with less methylation in LFH than in HFH animals, and vice versa for negative logFC for which LFH animals have more methylated strands than HFH animals. Significant windows are those with FDR-corrected *p* < 0.1

**Table 2:** Differentially-methylated windows between the selection lines

Chr	Window start (bp)	CpGs	logFC	Adjusted *p* value	Gene symbol	Gene description
1	121,257,001	4	− 1.33	9.52e − 02	*AP1S2*	Adaptor related protein complex 1 sigma 2 subunit [Source:HGNC Symbol;Acc:HGNC:560]
1	142,947,001	4	− 0.93	5.87e − 02	*NALCN*	Sodium leak channel, non-selective [Source:CGNC Symbol;Acc:12660]
1	174,158,001	7	− 0.90	9.52e − 02		
1	188,959,001	7	1.40	5.14e − 02	*DLG2*	Discs, large homolog 2 (Drosophila) [Source:CGNC Symbol;Acc:12965]
1	192,474,001	5	1.35	3.27e − 07	*CAPN5*	Calpain 5 [Source:CGNC Symbol;Acc:479]
2	73,558,001	3	− 2.75	8.12e − 02		
2	85,911,001	12	0.80	9.52e − 02		
3	9,149,001	7	1.11	3.22e − 02	*EHBP1*	EH domain binding protein 1 [Source:CGNC Symbol;Acc:6763]
3	53,750,001	7	1.31	2.73e − 02	*ARFGEF3*	ARFGEF family member 3 [Source:CGNC Symbol;Acc:10361]
3	106,780,001	5	1.70	4.83e − 03	*MTMR9*	Myotubularin related protein 9 [Source:CGNC Symbol;Acc:12476]
4	27,239,001	3	− 0.98	9.24e − 02		
5	30,875,001	3	− 0.80	3.22e − 02		
5	57,981,001	7	1.12	7.28e − 02	*C5h14orf166*	Gallus gallus chromosome 5 open reading frame, mRNA. [Source:RefSeq mRNA;Acc:NM_205369]
5	58,733,001	12	0.99	7.79e − 02		
11	16,608,001	16	− 1.29	5.91e − 02		
14	5,936,001	11	− 0.83	8.12e − 02		
15	6,629,001	22	1.13	3.22e − 02	*USP30*	Ubiquitin specific peptidase 30 [Source:CGNC Symbol;Acc:50370]
17	1,582,001	8	1.87	8.12e − 02	*MRPL41*	Mitochondrial ribosomal protein L41 [Source:CGNC Symbol;Acc:6639]
17	1,582,001	8	1.87	8.12e − 02	*PNPLA7*	Patatin-like phospholipase domain containing 7 [Source:CGNC Symbol;Acc:6632]
18	10,927,001	23	0.89	5.14e − 02	*ARMC7*	Armadillo repeat containing 7 [Source:CGNC Symbol;Acc:6023]
19	58,001	3	− 0.95	1.01e − 02	*DDX25*	DEAD-box helicase 25 [Source:HGNC Symbol;Acc:HGNC:18698]
20	11,544,001	11	1.15	3.22e − 02		
23	3,692,001	5	1.38	7.28e − 02	*GNL2*	Guanine nucleotide binding protein-like 2 (nucleolar) [Source:CGNC Symbol;Acc:1398]

Comparison between HFH and LFH red junglefowl individuals resulted in 22 differentially-methylated 1-kb windows at FDR-corrected *p* < 0.1. Of these, 13 aligned within genes or promoter regions of genes. The log fold changes (logFC) are based on the comparison between HFH and LFH birds, with HFH birds set as reference in the MEDIPS analysis

The random resampling did not result in significantly differentially-methylated windows in any of the 20 replicates [see Additional file 1: Table S1].

### Sex effects

Sex-specific effects on DNA methylation, estimated when comparing combined HFH/LFH females against HFH/LFH males, were much larger than the effects of selection. Comparison of DNA methylation patterns between sexes, with line excluded as a factor, revealed many differences, with over 2500 DM windows on the autosomes and almost 2000 on the sex chromosomes W and Z [see Additional file 2: Figure S1]. A more detailed analysis of the DM regions indicated that 79% of the windows were hypermethylated in males.

Successive selection of HFH and LFH birds seemed to result in large sex-specific effects in DNA methylation differences, with 66 windows being DM between HFH and LFH females (FDR adjusted *p* < 0.1) [see Additional file 3: Table S2]. Moreover, 51 DM windows were identified between HFH and LFH males (FDR adjusted *p* < 0.1). Comparison of DM windows identified within males and within females showed overlaps at only four windows, three of which were located on chromosome 7 and covered the expressed sequence tag (EST) Gga.15462, which was previously detected in testis mRNA in RJF and White Leghorn [48]. The methylation patterns for this EST region differed between selection lines and between sexes, i.e. it was highly methylated in HFH males and in LFH females. The fourth window was located on chromosome 1 in an intron of the *CAPN5* gene and was hypermethylated in HFH animals of both sexes.

### Functional annotation

Genes from the 22 windows that showed significant DM between HFH and LFH animals were extracted via Ensembl and used for gene ontology (GO) and pathway (WebGestalt) analyses. Comparison of genes associated with the DM windows between the two lines showed enrichment for a number of terms, but not after FDR correction (Table 3). The enriched terms were related to mitochondria, ion channel activity, ribosomes, and regulation of membrane potential. Due to the small size of the dataset, KEGG pathway enrichment analysis yielded no results. GO analysis of sex-specific line effects showed little overlap of enrichment in males compared to females but yielded terms related to DNA replication, the GABA receptor complex and chloride channel activity in males and terms related to transmembrane transporter activity, the synapse part, and neurotransmitter complex in females.

**Table 3:** Enrichment results for gene ontology (GO) analysis of differentially-methylated regions between selection lines across and within sexes

GO ID	GO term
Across sexes	
GO:0005261	Cation channel activity
GO:0003723	RNA binding
GO:0003924	GTPase activity
GO:0005759	Mitochondrial matrix
GO:0006412	Translation
GO:0006816	Calcium ion transport
GO:0042254	Ribosome biogenesis
GO:0042391	Regulation of membrane potential
Female	
GO:0044456	Synapse part
GO:0043197	Dendric spine
GO:0098878	Neurotransmitter complex
GO:0022803	Passive transmembrane transporter activity
GO: 0016597	Amino acid binding
GO:0008066	Glutamate receptor activity
Male	
GO:1902710	GABA receptor complex
GO:0045211	Postsynaptic membrane
GO:0005657	Replication fork
GO:0034776	Response to histamine
GO:0006312	Mitotic recombination
GO:0004520	Endodeoxyribonuclease activity
GO:0005254	Chloride channel activity
GO:0008094	DNA-dependent ATPase activity

Annotations for differentially-methylated windows were used to search for enrichment of terms in the groups, both for overall effects of selection objective and sex-specific changes

### Transcriptional effect

We examined the overlap between the DM windows identified here and the genomic regions that included genes that were previously reported as differentially expressed (DE) in the hypothalamus between the same selection groups and generation [38]. Genomic regions located 7.25 kb upstream from DE transcripts were evaluated in order to include promoter regions [28]. No direct overlaps were detected between DM and DE regions, even when including DM regions with a fold change higher than 1.5 and *p* < 0.05 before FDR correction. ConsensusPathDB analysis of these regions revealed that the DM genes *MRPL41* and *ARMC7* (Table 2) were significantly connected with the DE genes *MAEA* and *ANKRD1* that were detected by Bélteky et al. [38] through one intermediate node. DM windows within males and within females were also compared to previously reported DE genes, but neither sex showed any overlap between expression and methylation changes [38].

## Discussion

In this experiment, we studied the effects of bidirectional selection for tameness on the DNA methylome of the hypothalamus in RJF. After five generations of selection for high and low fear of humans, 22 genomic regions with differential DNA methylation in the hypothalamus were detected. The DM regions targeted several genes with functions that may be related to previously reported phenotypic selection responses [11, 36], such as metabolism and signaling. However, the number of significant windows before correcting for multiple-testing was much larger than 22 windows, with over 51,000 differently methylated windows observed at *p* > 0.05. This large reduction in significant windows after correcting for FDR may indicate that extensive variation exists within the groups for methylation patterns in regions that are not strongly affected by the selection process. Such naturally occurring inter-individual variation has already been reported in humans [49].

Among the 22 DM windows, none overlapped with DE genes reported for the same tissue of birds from the same populations, which agrees with other studies on RJF and domesticated White Leghorn in which no overlap between DM promoters and gene expression of the same genes was observed. This suggests that other epigenetic mechanisms are involved in regulating gene expression [28], or that DNA methylation changes in regions outside of promoter and gene regions, i.e. in intergenic regions, could affect transcription in a trans-acting fashion [50, 51], or that the DM regions affect genes that are not activated by any pathways at the life stage studied here. Furthermore, gene expression may be affected primarily by methylation changes of single CpG sites within a CpG-dense region, which may not be detected by MeDIP-seq [52].

Although we observed no direct overlap between DM and DE genes, GO analysis of genes included in DM windows revealed functions similar to those of the DE genes in the same group of animals. Interestingly, some DM regions were related to behavioral and metabolic pathways. Further research should confirm whether and how these pathways are affected by selection for tameness. However, based on the genes targeted by the DM regions between HFH and LFH animals, we can derive some hints. For example, *calpain 5* (*CAPN5*), which encodes a cytosolic cysteine protease that acts on signaling-related molecules and is involved in cell differentiation and proliferation [53], was reported to interact with nuclear receptors and thus to impact metabolism [54]. Mutations in the gene *sodium leak channel non*-*selective* (*NALCN*), which is involved in the control of neuronal excitability, can lead to speech impairment and intellectual disability in humans [55, 56]. The gene *DLG2*, which encodes a scaffold protein belonging to the membrane-associated guanylate kinase (MAGUK) family and is active at postsynaptic sites, is associated with neurodevelopmental disorders, schizophrenia and cognition in humans [57, 58]. Downregulation of the mitochondrial gene *deubiquitinase USP30* is reported to enhance degradation of damage mitochondria in human neurons, which is beneficial during neurological disorders [59]. The fact that several of the genes related to DM regions are associated with neuronal functions suggests that molecular mechanisms involved in the behavioral changes have emerged between HFH and LFH birds [60].

We compared the DM regions detected here with DM probes that were previously detected in the hypothalamus by Nätt et al. [28] between RJF and the domesticated White Leghorn, in order to test whether the same genes with a modified methylation status were found in the current experiment and during the domestication of White Leghorn. We did not identify any common genes between these two selection processes, which could be explained by the fact that the driver of domestication of White Leghorn was mainly selection for egg size, while in our selection lines the main driver was tameness. This suggests that different selection pressures generate distinctive sets of epigenetic changes, which in turn are related to specific phenotypic traits. This concept can also apply to the transcriptome, since we observed no concordance between previously published DE genes from the same birds as those used in this study and DE genes observed in the hypothalamus of RJF and White Leghorn [28, 38].

A possible explanation for the lack of overlap between DM regions in the current and previous studies of the chicken hypothalamus is that the observed differences may be related to differential genetic drift in HFH and LFH populations [28]. However, previous studies on gene expression differences in the same populations, along with an unselected line of RJF, indicated that the expression changes that occurred between the parental generation and the fifth selected generations resulted from the imposed selection and not from genetic drift [38, 39]. These results, together with the random resampling performed in the current experiment, strengthen the argument that the epigenetic changes observed are a consequence of the artificial selection imposed within a rather short period of time (5 generations), and not of genetic drift.

In addition to the DM regions detected between the HFH and LFH lines in this experiment, we found numerous sex-specific changes, which are consistent with previously reported results [61]. However, in our study, differences were larger than observed previously, because it covered the entire genome, in contrast to only the promoter regions in a previous study that analyzed DNA methylation using promoter arrays [61]. The largest sex-specific DM regions were associated to the genes *ZFR* on chromosome 1 and *MHM* on chromosome Z, which were both highly methylated in males. This large number of sex-specific differences in DNA-methylation suggests that the two sexes respond differently to selection pressures related to behavioral traits, in agreement with previous findings [60], but raising some interesting questions about underlying mechanisms. An interesting finding was that many of these sex-specific DNA methylation changes are on autosomes, which suggests that DNA methylation of autosomes plays a more important role than expected in gender-specific characteristics in the brain of vertebrates.

## Conclusions

We detected 22 DM regions by comparing hypothalamic DNA from RJF selected during five generations for high versus low fear of humans. Functional annotation of the genes associated with these DM regions showed that they are related to, for example, cellular metabolism and neural signaling, similar to what was previously reported in terms of gene expression differences for the same animals. Our results suggest that bidirectional selection for tameness involves epigenetic factors that can even differ in a sex-specific manner. Observation of divergent DNA methylation patterns in the hypothalamus after only five generations of artificial selection highlights the importance of epigenetic mechanisms, in addition to genetic composition, in evolutionary phenotypic variation that emerges in response to selection pressures. Future research should delve into the molecular mechanism involved in the emergence of somatic epigenetic differences during selection. Two options could explain this. One is that epigenetic differences are linked to genetic differences emerging during selection, as suggested by Verhulst et al. [13]. Another possibility is that selection on phenotypes would concomitantly select specific germ line epigenomes. Different germ line epigenomes in divergent selection lines would then influence somatic epigenomes later on in the ontogeny of each individual. Interestingly, we detected several sex-specific epigenetic changes on the autosomes, which raises the question about whether epigenetic differences have a role in gender-specific behavioral responses that are unrelated to sex chromosomes.

## Additional files

**Additional file 1: Table S1.** Randomized resampling to validate results, with distribution of HFH and LFH animals in each randomization. Three females and three males were selected at random from the pool of samples, while also allowing for resampling of the same individual more than once. Two groups were generated and analyzed using MEDIPS and the same parameters as those set for the treatment groups. The procedure was repeated 20 times. HFH—High Fear of Humans, LFH—Low Fear of Humans.

**Additional file 2: Figure S1.** Differentially-methylated (DM) windows between males and females. Differences in methylation between sexes for each window in the genome were visualized via a Manhattan plot. The red horizontal line indicates the threshold for significantly DM windows at *p* < 0.1 after FDR correction. Microchromosome labels have been filtered out for readability.

**Additional file 3: Table S2.** Sex-specific differentially-methylated (DM) windows. The MEDIPS analysis yielded 66 DM windows in males and 66 windows in females at *p* < 0.1. The log fold changes (logFC) were obtained by using highly fearful birds as reference in the MEDIPS analysis.
