# Supplementary figures and images for: Epigenetics and early domestication: differences in hypothalamic DNA methylation between red junglefowl divergently selected for high or low fear of humans
Source: Genet Sel Evol. 2018 Apr 2;50:13. doi: 10.1186/s12711-018-0384-z (PMC5880090; doi:10.1186/s12711-018-0384-z)

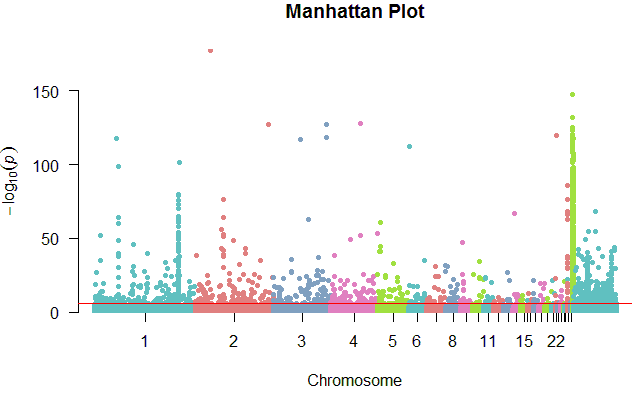

Supplement: Supplementary file 2 — Additional file 2: Figure S1. Differentially-methylated (DM) windows between males and females. Differences in methylation between sexes for each window in the genome were visualized via a Manhattan plot. The red horizontal line indicates the threshold for significantly DM windows at p < 0.1 after FDR correction. Microchromosome labels have been filtered out for readability. [file 12711_2018_384_MOESM2_ESM.bmp]
